# Ethnic variation of oral microbiota in children

**DOI:** 10.1038/s41598-020-71422-y

**Published:** 2020-09-08

**Authors:** Thyagaseely S. Premaraj, Raven Vella, Jennifer Chung, Qingqi Lin, Panier Hunter, Kori Underwood, Sundaralingam Premaraj, Yanjiao Zhou

**Affiliations:** 1 College of Dentistry, University of Nebraska Medical Center, Lincoln, NE USA; 2 Department of Medicine, UCONN Health Center, 263 Farmington Ave, Farmington, CT 06030 USA; 3 The Jackson Laboratory for Genomic Medicine, Farmington, CT USA

**Keywords:** Microbiome, Oral diseases

## Abstract

Despite widely used preventive measures such as sealant programs to control caries prevalence, disparities are seen among ethnic groups. Supragingival plaque harbors hundreds of bacterial species, playing a significant role in oral health and disease. It is unknown whether the ethnic variation influences the supragingival microbiota in children. In our study, variations in microbiota of the supragingival plaque was investigated from 96 children between 6 and 11 years old in four ethnic groups (African American, Burmese, Caucasian, and Hispanic) from the same geographic location by 16S rRNA gene sequencing. We found that the microbial alpha and beta diversity of supragingival microbiota significantly differed between ethnic groups. The supragingival plaque microbiota had the most complex microbial community in Burmese children. Within-group microbiota similarity in Burmese or Caucasian children was significantly higher than between-groups similarity. We identified seven ethnic group-specific bacterial taxa after adjusting for dental plaque index, decayed missing filled teeth (DMFT) and the frequency of brushing. Children with high plaque index and high DMFT values were more similar to each other in the overall microbial community, compared to low plaque index or low DMFT groups in which inter-subject variation is high. Several bacterial taxa associated with high plaque index or high DMFT were ethnic group-specific. These results demonstrated that supragingival microbiota differed among ethnicity groups in children.

## Introduction

The oral microbiota is composed of hundreds of bacterial species^1^, playing a significant role in oral health and disease. In addition to environmental factors such as diet^2,3^, birth mode^4^ and geographical location^5^, host genetics^6–8^ also plays an important role in configuring the microbiome community structure and determining pathogenic species associated with dental caries. These findings are in line with clinical observations that susceptibility to dental caries and periodontal diseases differs by ethnicity^9–11^. This ethnic disparity to oral diseases still holds true after controlling for socioeconomic, dietary, and other environmental factors^12^, raising the question of whether there is an ethnicity-specific microbiome that is associated with oral health and diseases.

Several studies have investigated the oral microbiome difference among different ethnicities. In a large cohort study, 32 differential bacterial taxa were identified in oral rinse samples from adults with African ancestry and European ancestry^13^. In the same study, significantly higher microbial diversity was also found in the African ancestry group compared to the European ancestry group. In another study, ethnicity-specific microbiota signatures were found in subgingival plaques from African Americans and Caucasians^14^. Comparison of the saliva microbiome among native Alaskans, Germans and Africans revealed distinctive microbiome compositions in each group as well as a few core microbes that were shared by all studied subjects^5^. Although this study represented three ethnic populations, the three groups also came from different climate zones among which lifestyle may differ, thus potentially confounding the conclusion that the microbiome was specific to either ethnicity.

Notably, studies on the association of the microbiome with ethnicity have been focused on the adult populations only. No study to date has compared the oral microbiome in children of different ethnicities. Right after birth, bacteria start to colonize the oral cavity^15^. The microbial community becomes more diverse over the first several years of life and continues to vary through the development of teeth, and finally become stable around adulthood^16–18^. Early oral microbiota development has a significant impact on oral health and diseases in adulthood^19^. Understanding the oral microbiota difference in children in different ethnicities including the ethnic minority will allow us to gain deep insight into the factors that might drive oral health disparities. In this study, we collected supragingival samples from 96 children, 6–11 years old from Caucasian, African American, Hispanic and Burmese ethnic groups in the same geographical location in Nebraska to ask the following questions: (1) whether the supragingival microbiota in children differs by ethnicity and (2) whether ethnicity-associated microbial variation contributes to the dental health disparities, specifically, the decayed missing and filled teeth (DMFT) and plaque index (PI). The outcome of the study will expand our knowledge of ethnic association of oral microbiota in caries risk and bring to light the need for group-targeted or individualized prevention strategies.

## Materials and methods

### Study population

This study was approved by the University of Nebraska Medical Center Institutional Review Board (IRB #199-15-EP). All methods were carried out in accordance with relevant guidelines and regulations. All participants and their caregivers gave written informed consent to participate. Caucasian, African American, Hispanic and Burmese are the four most common ethnic groups at the pediatric dental clinic of University of Nebraska Medical Center. Burmese and Hispanic children included in the study were first-generation immigrants. Patients who attended the pediatric dental clinic of UNMC for regular dental care, between the age of 6–11 years, had second primary molars without full coverage restoration by stainless steel crowns, were not under any restricted dietary patterns due to food allergies or gastroenterological condition that may have restricted certain food intake, were healthy and not medically compromised were recruited for this study. Plaque index (PI), and past lesions (already restored teeth due to caries) and present carious lesions (caries detected on the day of examination) were documented by a single pediatric dental resident. All patients were residents of Nebraska. PI was measured according to the Loe plaque index^20^ and the cumulative score was obtained and categorized as low risk (PI ≤ 1.5), and high risk (PI > 1.5), taking into account all other oral hygiene indexes described in the literature^21^. The sum of decayed missing and filled teeth (DMFT) was calculated for both primary (dmft) and permanent dentitions (DMFT). The total decayed missing filled teeth in both primary and permanent dentition was represented as DMFT(t) throughout the paper. All missing teeth were extracted due to caries in this study group. Currently, there is no standard criterion to categorize DMFT in to high and low risk groups in children. At the age of 6–11, it is expected at least 24 teeth will be in the oral cavity. In our analysis, if fewer than 25% of existing teeth (6 teeth) in a patient was decayed, missing, or filled, they were considered low risk (DMFT(t) ≤ 6) and if greater than 25% as high risk (DMFT(t) > 6). Information about oral hygiene habits (brushing frequency) and dietary habits (sugar and sugary product intake) was obtained by a questionnaire completed by the present legal guardian. The caries risk was evaluated based on American Academy of Pediatric Dental Guidelines. All participants were catagorized under the low socio-economic group in this study.

### Sample collection, DNA extraction, and sequencing

Supragingival plaques on all present tooth surfaces (buccal and lingual) were collected using a dental scaler, placed into a microfuge tube with PBS solution (phosphate-buffered saline), and stored at −80 °C until further analysis. DNA was extracted utilizing a mini DNA kit (QIAGEN) according to the manufacturer’s instructions. To break gram-positive cell walls, in addition to the lysis buffer, a mixture of beads containing two-thirds of 1.0 mm zirconia/silica beads and one-third of 0.5 mm zirconia/silica beads were used. All samples were concentrated down using a VWR Savant Speedvac to a concentration of about 5 ng/μl. The V3-V4 regions of 16S rRNA genes were amplified by PCR per the Illumina protocol ‘16S Metagenomic Sequencing Library Preparation’ (https://support.illumina.com/downloads/16s_metagenomic_sequencing_library_preparation.html). The amplicon size was verified by Agilent BioAnalyzer 2,100 DNA 1,000 chip with an expected size of ~ 550 bp. An additional PCR reaction was performed to add Illumina sequencing adapters and dual-index barcodes using the Nextera XT Index kit. 300-bp paired-end sequencing was performed on the Illumina MiSeq platform using the Miseq V3 600 cycle kit at The University of Nebraska DNA Sequencing Core. Negative controls including the extraction controls and PCR controls were included in the sample preparation and sequencing process.

### Sequencing, data processing, and statistical analysis of the microbiota data

Sequencing data processing and OTU generation were performed using USEARCH pipeline as we have done recently in Integrative Human Microbiome Project^22^. In brief, sequencing reads with an average quality score less than 25 were filtered out using Trimmomatic (v0.38). High quality paired-end reads were merged using Flash (v1.2.11). Merged reads were subjected to chimera removal using UCHIME (v4.2.40) and formed OTU representative sequences at 97% threshold. Representative OTUs that contain ≤ 4 reads were removed. The final representative sequences were further classified by RDP classifier (v2.11) against Greengenes database with 0.8 confidence value as cut-off. To assign OTUs of interest to specific taxonomical names at species level, the representative OTU sequence was blasted to NCBI 16S rRNA gene database and the top 1 hit from the blast was considered the closest taxa to the query OTUs when the identity of query sequencing is > 97% of the reference sequence. If different taxa were identified with the same bit scores, manual inspection was performed to choose the taxon based on known knowledge of the taxa. Taxa that were present in DNA extraction control and PCR controls which were potential contaminations were removed for downstream analysis from the sequenced samples.

Microbiota diversity was determined by Richness and Shannon Diversity as previously described^23^. Statistical difference of microbial diversity was performed using Kruskal Wallis testing in > 2 group comparisons, followed by Dunn’s test for between group comparisons. Wilcoxon Rank testing was performed in 2 group comparisons. Beta diversity, determined by Bray–Curtis dissimilarity was calculated as we have done previously^23^. Within and between group comparisons of microbial Beta diversity was conducted by a two-sample t-test. Microbiota variation between subjects was illustrated using Principle Component Analysis. Permutational multivariate analysis of variance (PERMANOVA) was used to determine the statistical difference of the overall microbiota composition and abundance between groups, and covariates including PI, DMFT(t) and brushing frequency were included in the model. Differences in the relative abundances of specific taxa between the groups were determined with DESeq2^24^. P values were adjusted based on False Discovery Rate in multiple comparisons. Adjusted p-value ≤ 0.05 was considered statistically significant. Co-occurrence and co-exclusive analysis were performed using SparCC^25^ to first identify the positive and negative correlation between taxa and plaque index, followed by visualization through network analysis with Spring layout to identify a group of interconnected taxa that are associated with plaque index. Correlation coefficients > 0.4 or < -0.4 and between taxa or taxa with plaque index were plotted. All the statistical analyses were performed in R (version 3.5.0).

### Ethical standard

This study was approved by the University of Nebraska Medical Center Institutional Review Board (IRB 199-15-EP). All participants and their caregivers gave written informed consent to participate.

## Results

### Demographics of the studied cohort

Supragingival samples were collected from 96 children (ages 6–11 years) in four different ethnicities including African American (n = 26), Burmese (n = 19), Caucasian (n = 26) and Hispanic (n = 25) (Table 1). There was no significant difference in gender distribution across ethnic groups in our cohort. Burmese children have significantly lower frequency of daily brushing, compared to the other three ethnic groups (p = 0.03 by Kruskal–Wallis test). Plaque was most prevalent in Burmese children (18/19 = 94.7%), followed by Hispanic children (17/25 = 68%), African American (15/26 = 57.6%) and Caucasian (14/26 = 53.8%) (p = 0.01 by Chi-square test). DMFT(t), which represented the caries experience of an individual also showed a significantly different prevalence across ethnicities (p = 0.008 by Chi-square test). Burmese children had the highest prevalence (17/19 = 89.5%) of high number of DMFT(t), followed by Hispanic children (16/25 = 64%), and African American and Caucasian (12/26 = 46.2%). Although children with high PI tended to have high number of DMFT(t), 25% (16/64) of the children with high PI had low number of DMFT(t), and 28.1% (9/32) of children with low PI had high number of DMFT(t). Dietary habit such as sugar intake was also compared. All children in the study had high level of sugar consumption in terms of amount and frequency (Supplementary Table S1) based on American Academy of Pediatric Dental Guidelines^26^ .

**Table 1:** Demographics of the study cohort.

Ethnicity	Participants (n)	Age (years) (m ± sd)	Gender (n)		Daily brush frequency (m ± sd)*	Plaque index (n)**		DMFT(t) (n)***	
			Female	Male		Low	High^#^	Low	High^#^
African American	26	7.6 ± 1.5	10	16	1.0 ± 0.8	14	12 (46.2%)	7	19 (73.1%)
Burmese	19	7.7 ± 1.3	7	12	0.7 ± 0.8	2	17 (89.5%)	12	7 (36.8%)
Caucasian	26	7.7 ± 1.5	12	14	1.2 ± 0.6	14	12 (46.2%)	22	4 (15.4%)
Hispanic	25	7.6 ± 1.3	14	11	1.4 ± 0.6	9	16 (64%)	16	9 (36.0%)
Total	96	7.7 ± 1.4	43	53	1.1 ± 0.7	39	57 (59.4%)	57	39 (40.6%)

*p = 0.03; **p = 0.01; ***p = 0.008.

^#^Proportions of high plaque index or high DMFT(t) are indicated in the bracket for any given ethnicity.

### Ethnic groups contribute to the overall microbiota variation

**Figure 1:**
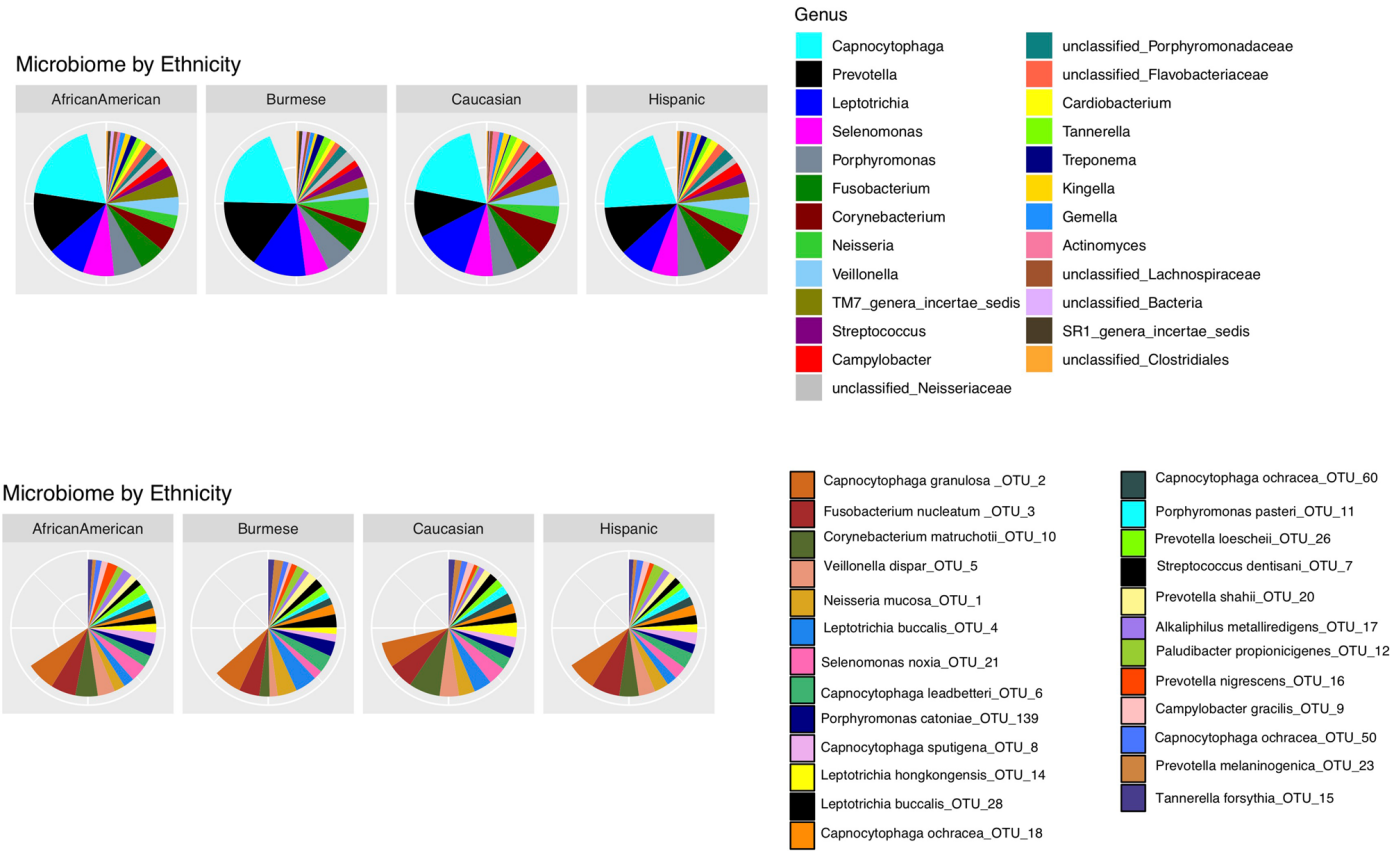
Supragingival plaque microbial compositions and abundances at genus and OTUs level in four ethnic groups. Top 25 most abundant genera or OTUs were plotted. The blank space in the pie chart represents the rest of the genera or OTUs. *Capnocytophga* is the most abundant genus. *Capnocytophga granulosa* is the most abundant species in our study cohort.

The V3-V4 region of 16S rRNA gene sequencing yielded 3.2 million reads in total, with an average of 30,000 reads ± 13,000 reads/sample. We have identified 218 OTUs from 64 genera. The predominant genera *Capnocytophaga, Prevotella, Leptotrichia, Selenomonas, Porphyromonas, Fusobacterium, Cornybacterium, Neisseria, Veillonella, TM7-genera-incertae-sedis* were commonly found with similar representation regardless of ethnicity (Fig. 1).

**Figure 2:**
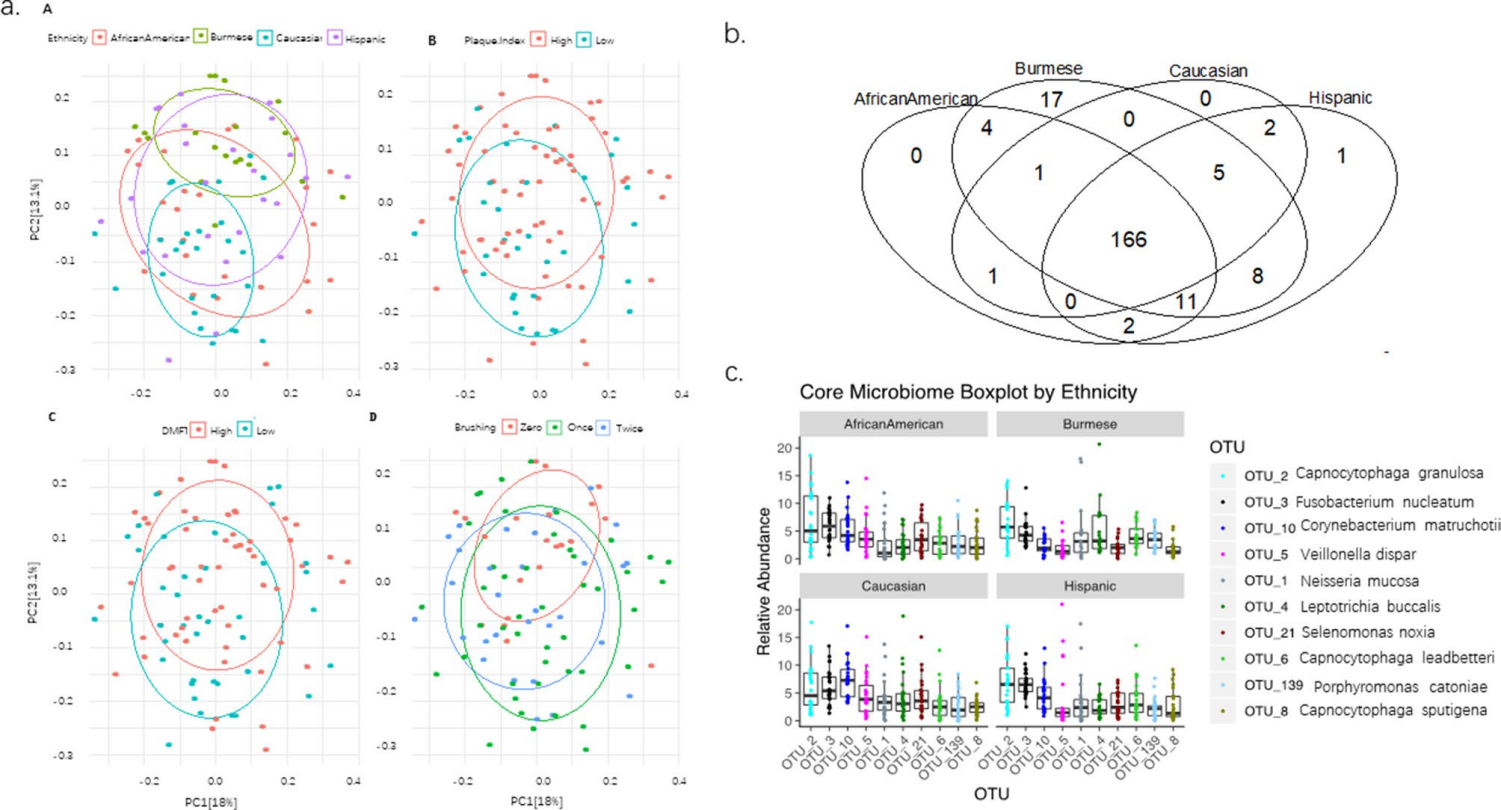
Overall microbial comparison across four ethnicities. (**a**) Principle component analysis (PCA) of the supragingival microbiota by ethnicity, PI, DMFT(t) and brushing. Groups in each PCA are displayed by confidence ellipses of one standard deviation with different colors. (**b**) Shared and unique OTUs among four ethnic groups. (**c**) The abundance difference of the top10 most abundant core OTUs shared by all ethnic groups.

To determine what drives the microbiota variation in our study cohort, we tested the contribution of demographic factors in Table 1 to the microbiota variation using Permutational multivariate analysis of variance (PERMANOVA). The microbiota had significant variation by ethnicity (p = 0.001), plaque index (p = 0.03), DMFT(t) (p = 0.08) and brushing (p = 0.04) when these variables were tested individually. Ethnicity accounted for the highest total variance (8.5%) of the microbiota, and maintained significance after adjusting for plaque index, brushing and DMFT(t) (p = 0.001) (Fig. 2a). Pair-wise comparison of the overall microbiota community between ethnicities, the microbiota between African American and Hispanic ethnicities showed no statistical difference, while the rest of the comparisons were all statistically different (p < 0.05). Overall microbiota community structure did not differ by PI, brushing frequency or DMFT(t) after adjusting for ethnicity.

To identified the taxa that are unique and common to the four ethnic groups, a Venn diagram was created (Fig. 2b). 166 (76.1%) out of 218 OTUs were shared by all four ethnicities (Fig. 2b). While there were only a few unique microbes from African American and Hispanic children, 17 OTUs were unique to Burmese children, when all samples were subsampled at the same sequencing depth. On average, these unique OTUs accounted for 0.6% of the total microbial community. One of these OTUs belong to the genus *Sneathia* and was the most abundant OTU (0.1% on average) among Burmese children. Addtionally, the 17 unique OTUs were present in ~ 50% of the Burmese children, suggesting it is likely that they are common bacteria in the Burmese community. Figure 2c shows the top 10 most abundant core microbes. OTU_2 *Capnocytophaga* was the abundant bacterial taxon in both Burmese and Hispanic children. OTU_3 *Fusobacterium* and OTU_10 *Corynebacterium* were the most abundant OTUs in African American and Caucasian children, respectively.

### Alpha and beta diversity of the microbiota in Burmese children and children with high PI and high DMFT(t)

**Figure 3:**
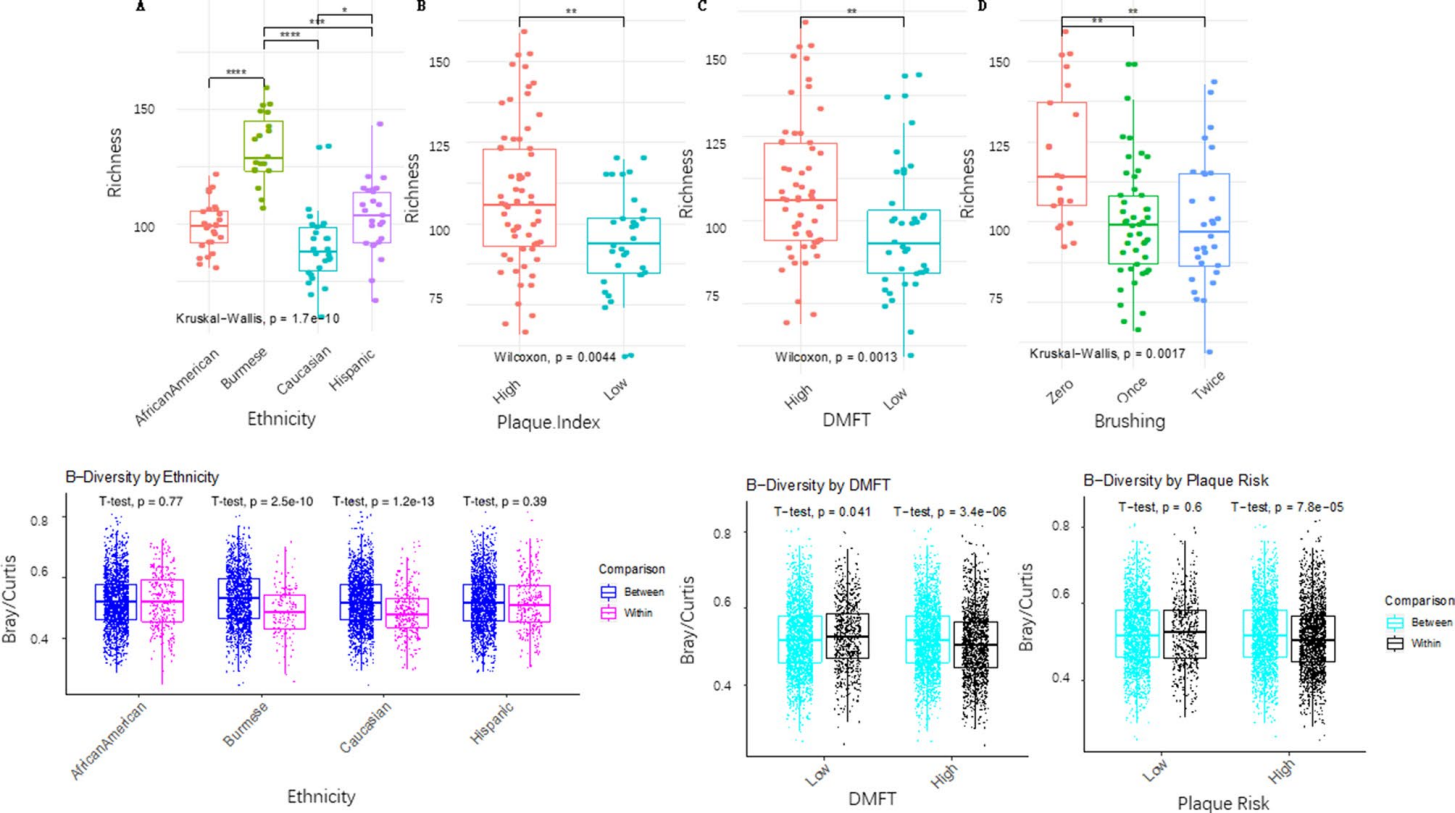
Alpha and Beta diversity among different ethnic, DMFT(t), and plaque index groups. (**a**) Richness difference. A significantly higher richness was found in children from the Burmese group, high plaque index group, high DMFT(t) group and high brushing frequency group. The degree of statistical significance after multiple comparison adjustment is indicated as below: ****p < 0.0001, ***p < 0.001, **p < 0.01, *p < 0.05. (**b**) Between and within the group microbial dissimilarity measured by Bray–Curtis dissimilarity. Burmese and Caucasian children were more similar within their own ethnicity, while the microbial similarity within African American and Hispanic children were similar compared to other ethnic groups. Children with high DMFT(t) or high plaque index have more similar microbiota within-group than to children in low and high groups.

Alpha diversity including richness and Shannon diversity is an important indicator of the microbial complexity and has been associated with health in many diseases^27^. Bacterial richness was significantly different among the four ethnicities by the Kruskal–Wallis test (p = 1.7e-10). Burmese and Caucasian children harbored the highest and lowest number of different bacteria in their supragingival plaque, respectively. Richness in the Burmese group was significantly higher than that of the other ethnicities by the Dunn test (p < 0.0001, Fig. 3a). The richness of the Caucasian group was significantly lower than any other ethnicity (p < 0.05). Richness was not significantly different between Hispanic and African American groups (p > 0.05). Shannon diversity showed similar findings (data not shown). Richness was also significantly higher in children with high PI (p = 0.004), high number of DMFT(t) (p = 0.001) or high brushing frequency (p = 0.002). To determine whether different microbial diversity in different ethnicities is independent of PI, DMFT(t) and daily brush, we performed a Generalized Linear Regression (GLR). GLR analysis showed that ethnicity remained statistically different between Burmese and the rest of the three groups (p < 7.94e−08), between African American and Caucasian (p = 0.01), after adjusting for PI, DMFT(t) and brushing frequency.

Beta diversity measures the inter-subject variation of the microbiota. To understand the variation of microbiota within and between ethnic groups, with PI and DMFT(t), we computed the Bray–Curtis dissimilarity between and within compared groups. Interestingly, subject variation was significantly lower within Burmese or Caucasian groups than between ethnic groups (Fig. 3b). This was in contrast with African American and Hispanic groups in which no statistical difference was found between within-group similarity and between-group similarity of the microbiota. In addition, children in high PI groups or high DMFT(t) groups were more similar to each other than to low PI groups or low DMFT(t) groups.

### Specific taxa associated with ethnicity

**Figure 4:**
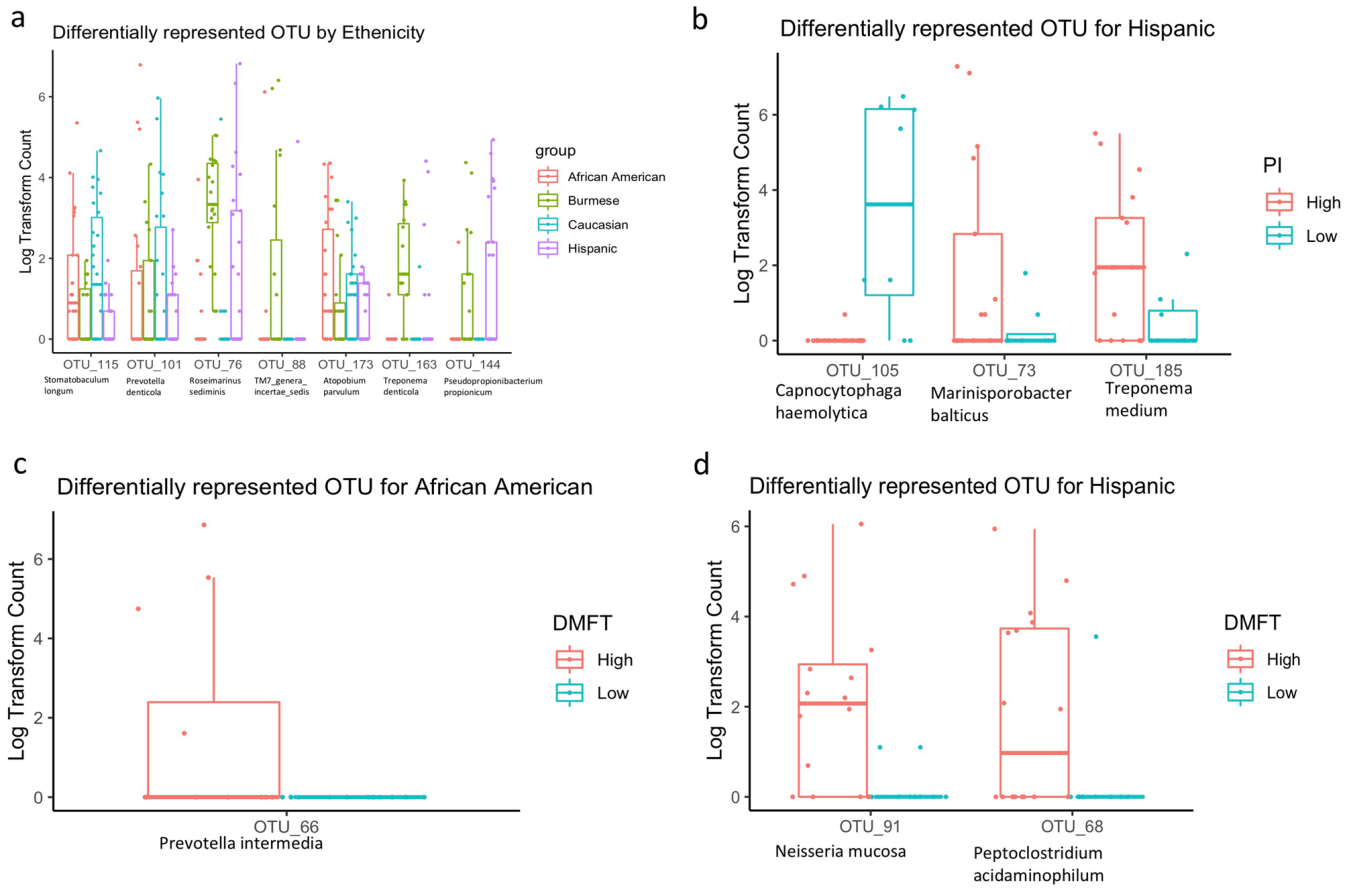
Specific OTUs of supragingival plaque that differs in children from different ethnicity, high/low plaque index and high/low DMFT (t). (**a**) OTUs that were differentially represented in four ethnic groups after adjusting for plaque index and DMFT(t). (**b**) OTUs that were differentially represented in Hispanic children with high plaque index. (**c**) OTUs that were differentially represented in African American children with high DMFT(t). (**d**) OTUs that were differentially represented in Hispanic children with high DMFT(t).

To identify specific taxa that are significantly different among ethnic groups, we performed DESeq2 using original count data after adjusting for PI, DMFT and daily brushing frequency. As shown in Fig. 4a, seven OTUs were differentially represented among four ethnic groups (adjust p < 0.05). OTU_76 from unclassified_*Bacteroidetes* (closest to *Roseimarinus sediminis* by blast the reads to 16s rRNA gene database in NCBI), OTU_88 from TM7_genera_*incertae_sedis* and OTU_163 *Treponema* were most dominant in Burmese children. OTU_101 *Prevotella* and OTU_115 unclassified_*Lachnospiraceae (*closest to *Stomatobaculum longum*) were most dominant in Caucasian children. OTU_173 *Atopobium* and OTU_144 unclassified_*Propionibacteriaceae (closest to Pseudopropionibacterium propionicum*) showed the highest relative abundance on average in African American children and Hispanic children, indicating that there are differentially abundant bacteria by ethnicity in our cohort.

To examine whether the high plaque and DMFT(t) associated taxa are specific to any ethnicity, we compared the taxa differences in high and low PI or DMFT children within each ethnicity. However, we did not compare differences in Burmese children because the vast majority of them were in the high plaque and high DFMT(t) group. OTU_73 and OTU_185 associated with high plaque index were only found in Hispanic children and not African American nor Caucasian children, suggesting that different ethnicities may potentially harbor unique PI-associated bacteria (Fig. 4b). This result is consistent with the finding that ethnicity affects the overall microbial community structure, as demonstrated in Fig. 2a.

OTU_66 was over-represented in African American children with high DMFT, but not statistically enriched in high DFMT(t) groups in Hispanic and Caucasian children (Fig. 4c). OTU_91 and OTU_68 were only over-represented in Hispanic children with high DMFT (Fig. 4d). We did not find any taxa statistically associated with DMFT(t) in the Caucasian group, although the prevalence of high and low DMFT(t) children was similar to that of African American children.

### Microbiota co-occurrence and high plaque index

Lastly, we identified high plaque and high DMFT(t) associated bacteria by DESeq analysis after adjusting for ethnicity. The relative abundances of eight OTUs were significantly higher in high plaque children (Supplementary Fig. 1a). This included two different species of *Treponema* (OTU_84*-T. denticola* and OTU_185*-T. medium*), two species of *Prevotella* (OTU_95*-P. pleuritidis* and OTU_101*-Prevotella denticola*), and taxa identified as *Neisseria*, *TM7*, unclassified *Bacteriodetes*, and *Lachospiraceae*. Only one OTU from *Prevotella *(*OTU_66 Prevotella intermedia*) was found to be significantly higher in children with high DMFT (Supplementary Fig. 1b). Interestingly, the taxa associated with high PI did not overlap with the taxa associated with high DMFT(t). This is not surprising as not all the high PI children had high DMFT(t) (Table 1), although high PI is considered a risk factor for DMFT(t).

Considering the oppositional and symbiotic relationships among the microbiota in an oral ecological niche, we further built the microbial correlation network among the OTUs with at least 0.5% of relative abundance using SparCC to identify groups of microbes that were associated with high PI. As shown in Supplementary Fig. 1c, OTU_159 *Prevotella histicola* was positively correlated with PI. Interestingly, OTU_159 was also positively correlated with OTU_55 *Alloprevotella tannerae* (r^2^ = 0.43) and negatively correlated with OTU_8 *Capnocytophaga sputigena*, even though OTU_8 and OTU_55 were not directly associated with PI. In addition, OTU_9 *Campylobacter gracilis* was negatively correlated with PI. OTU_9 was positively correlated with seven other OTUs across different genera.

## Discussion

Effectively reducing oral disease burden requires a better understanding of its determinants. To address the health disparities in the prevalence of dental caries, our study determined the microbiota profile of the supragingival plaque among 96 children from different ethnicity groups. These children aged 6–11 years are in a developmental stage called mixed dentition, a transitional stage when primary teeth are being replaced by permanent teeth. Previous study has shown that 90% of the oral microbial composition and functionality were similar between primary and permanent dentitions^28^. However, specific microbes are still under development at this age. For instance, the saliva in deciduous dentition harbored higher abundance of Proteobacteria than those in mixed and permanent dentition, and the relative abundance of *Prevotella* increased from deciduous, mixed to permanent dentitions^17^. In our study, supragingival microbiome was dominated by *Capnocytophaga* (18.9% of total bacteria), *Prevotella* (12.5%) and *Leptotrichia* (10%) from all four ethnic groups. *Capnocytophaga* is a normal resident oral flora in children and adults, but its prevalence was higher in people with periodontitis or gingivitis^29,30^. The high abundance of *Prevotella* and *Leptotrichia* in our study is consistent with the finding in the supragingival microbiome profile of healthy Chinese children^31^, but inconsistent with others. For example, one study of children from Philadelphia (no ethnicity data was included) reported that the most abundant bacterial genus from the supragingival plaque was *Actinomyces* (~ 36% of total bacteria)^32^ while a large cohort study of Australian children of a similar age range reported that *Veillonella* was the most abundant genera in their dental plaques (no ethnic data mentioned in the paper)^6^. Geographical location, diet, climate, host genetics or different study approaches such as DNA extraction methods may all play a role in the observed differences to a certain extent.

Our study included children from four ethnicity groups: Caucasian, African American, Hispanic and Burmese groups. After adjusting for the presence and/or quantity of plaque as well as daily brushing frequency, we found that the overall supragingival microbiota profile remained different by ethnicity, suggesting that ethnicity itself may act as an independent variable that potentially affects the microbiome profile. Ethnicity accounted for the highest total variance of the supragingival microbiota, compared to PI, DMFT (t) or brushing frequency. We also found alpha and beta diversities differed by ethnicity. Interestingly, the supragingival microbiota in Burmese or Caucasian children were more similar to their own ethnicities, reinforcing the idea that the microbiome profile is ethnicity specific. No study to date has investigated the oral microbiome in Burmese children. Our study for the first time revealed the strikingly high prevalence of PI, lowest daily brush frequency and highest bacterial diversity for this understudied group. Our finding that African American had higher microbial diversity than Caucasians is consistent with a recent large cohort study showing that African American adults had a higher bacterial richness in oral washes than those from European ancestry^13^. By contrast, another study indicated that African American adults had the lowest bacterial diversity in subgingival plaques while Chinese and Caucasian adults had the highest diversity^14^. However, the sequencing depth was relatively shallow in the latter study, which may prevent an accurate estimate of the microbial diversity. Despite the technical difference, many confounding factors as described above can potentially affect the microbiome makeup. This makes it extremely challenging for any cross-study comparison. Our finding concluded that children, with mixed dentition from different ethnic groups, like adults, may have an ethnic-associated oral microbiome.

Although the mechanism for these ethnicity-dependent microbiome difference is not completely understood, genetic factors^6–8^, dietary patterns^2,3^, disease conditions^33^ and other practices could all contribute. All study participants in our cohort were low-income residents of Nebraska. The Burmese children are first-generation immigrants living in the Nebraska area. The Burmese group may exhibit differences in lifestyle when it comes to dietary practices, oral hygiene practices, access to dental health care and ignorance of the value of primary teeth, all of which may contribute to the high bacterial richness and different microbial profile. Acculturation has been shown to impact oral health^34,35^; it will be interesting to test whether and to what extend acculturation alters the oral microbiome or ethnicity related microbiome signature identified in these Burmese children in future.

Carbohydrate consumption was found to affect microbial community structure^36,37^. High consumption of fermentable carbohydrates was associated with reduced bacterial diversity in occlusal biofilms^37^. In our study, all children had high levels of sugar consumption in terms of amount and frequency and were identified as high risk for caries by the pediatric dentist. Thus, sugar consumption was not a confounding factor to our observed results. Future studies including a comprehensive food record will be essential to elucidate the contribution of diet on the oral microbiome variation by ethnicity.

Twin studies showed that host genetics was one determinant of supragingival microbiome composition^7,8,33^. Genes coding for ATP-binding cassettes and protein synthesis seem to have an impact on the abundance of *Prevotella*, *Pasteurellaceae* and *Leptotrichia*^6^. However, it is still controversial whether caries-associated bacteria are influenced by genetic factors^6^. Our study identified several bacterial OTUs from *Treponema* and *Capnocytophaga* that were associated with PI and DMFT(t) in Hispanic children but not in other ethnic groups. The lack of association of these bacteria with PI or DMFT(t) in other groups may reflect the small sample size after stratification. Future studies with larger sample sizes and strain level characterization are needed to verify the results. If confirmed, group-specific preventive strategies for caries would be of importance to restore oral health and to eliminate the currently existing health disparity.

PI is an indicator of poor oral hygiene^38^ and associated with caries risk^39^. We found significantly higher bacterial richness in the high PI group, indicating that higher PI is associated with overgrowth of different bacteria. Thus, despite altered relative abundance of specific microbes, emerging new bacteria represent another aspect of imbalance in the supragingival microbiome. At the whole community level, we found that the microbial beta diversity of children in high PI groups or high DMFT(t) groups were more similar to each other than those in low PI or low DMFT(t) groups. Namely, healthy oral microbiomes in children were more heterogenous while microbiome in an oral disease condition were more homogenous across individuals. Significant increase of PI or DMFT(t) associated bacteria may drive the convergence towards a more similar microbial community in those children. Despite increased alpha diversity in caries, our study additionally identified interesting changes in beta diversity. The latter offers a novel insight into the microbial changes in caries or DMFT(t) at the overall microbial community level.

Finally, in addition to the association of certain species with high PI or with high DMFT(t), this study also found that there was a complicated bacteria-bacteria interaction and complex associations between bacteria and PI. This network analysis complemented the two group comparisons approach (high vs low PI groups) that identifies specific single microbes differing between high and low PI groups. It views the oral microbiome as an interactive entity and reinforces the idea that caries is associated with changes of multiple members in the microbiome community. We have identified additional microbes including OTU_159_*Prevotella* and OTU_9_*Campylobacter* that were positively and negatively correlated with PI. Some *Prevotella* species were shown to be highly abundant in caries^40^ while some *Campylobacter* species were significantly reduced in severe caries in young children^41^. These bacteria formed multiple correlations with other bacteria that may potentially relate to caries. Our understanding of the etiology of caries has been evolving from the early specific plaque hypothesis to the non-specific plaque hypothesis and now to the more recent ecological hypothesis and keystone-pathogen hypothesis^42^. The co-occurrence and mutual exclusivity of the oral microbiome in our study strongly suggests a polymicrobial synergy in oral health and disease. Identifying the presence, absence, and the co-existence of bacterial species in a polymicrobial environment is a crucial step in the process of understanding their contributions to the oral niche. Until we bridge the gap between metagenomics and functional genomics, the intricate relationships within a microbial population cannot be fully understood.

A strength of our study is the involvement of four different ethnicities, including the Burmese whose oral microbiome has not been studied to date. In addition, our work represents the first study focused on the children’s supragingival microbiome in different ethnicities. Previous studies have identified ethnicity-specific microbes in adults^13,14^. Our work highlights that the early oral microbiome had already exhibited ethnicity associated signature. The children participated in the study were all from the same region of Nebraska and belong to low-economical group, therefore the microbiome difference we observed was less likely to be confounded by these factors that potentially impact the microbiome composition. There are several limitations in our study, including a relatively small sample size and a cross-sectional study design. This pilot study identifies potentially interesting ethnic-specific microbiome signatures and lays a conceptual foundation for large and longitudinal studies in future.

In summary, we found that supragingival microbiome varied by ethnicity in children. This raises a possibility that variation of the oral microbiome may contribute to ethnicity disparity of oral health. Functional characterization of oral microbiome and longitudinal evaluation of signature bacteria of different ethnicities in future will shed light on the complex interplay between environment and host genetics in controlling the oral microbiome ecosystem.

## Supplementary information

Supplementary Table S1.

Supplementary Figure S1.

## Data Availability

The raw sequencing data from 16S rRNA gene sequencing used in this study are available in short reads archive (SRA) database with accession number PRJNA555622.
