# Author Correction: Ethnic variation of oral microbiota in children

**DOI:** 10.1038/s41598-021-95052-0

**Published:** 2021-07-29

**Authors:** Thyagaseely S. Premaraj, Raven Vella, Jennifer Chung, Qingqi Lin, Hunter Panier, Kori Underwood, Sundaralingam Premaraj, Yanjiao Zhou

**Affiliations:** 1 College of Dentistry, University of Nebraska Medical Center, Lincoln, NE USA; 2 Department of Medicine, UCONN Health Center, 263 Farmington Ave, Farmington, CT 06030 USA; 3 The Jackson Laboratory for Genomic Medicine, Farmington, CT USA

Correction to: *Scientific Reports* 10.1038/s41598-020-71422-y, published online 08 September 2020

The original version of this Article contained an error in the spelling of the author Hunter Panier which was incorrectly given as Panier Hunter.

The original Article and accompanying Supplementary Information files have been corrected.

