# Supplementary material for: Ethnic variation of oral microbiota in children
Source: Sci Rep. 2020 Sep 8;10:14788. doi: 10.1038/s41598-020-71422-y (PMC7478955; doi:10.1038/s41598-020-71422-y)
Supplement: Supplementary file 1 — Supplementary Table S1. [file 41598_2020_71422_MOESM1_ESM.docx]

**Ethnic Variation of Oral Microbiota in Children**

Thyagaseely S Premaraj^1^, Raven Vella^2^, Jennifer Chung^2^, Qingqi Lin^2^, Panier Hunter^2^, Kori Underwood^1^, Sundaralingam Premaraj^1^, Yanjiao Zhou^2,3^

**Affiliations:**

^1^College of Dentistry, University of Nebraska Medical Center, Lincoln, NE, USA

^2^ Department of Medicine, UCONN Health Center, Connecticut, USA

^3^The Jackson Laboratory for Genomic Medicine, Connecticut, USA

**Corresponding Author:**

Yanjiao Zhou, M.D, Ph.D

Department of Medicine, UCONN Health Center, Connecticut, USA

263 Farmington Ave, Farmington, CT 06030

**Phone: 860-679-6379**

**Email: yazhou@uchc.edu**

**Supplementary Table S1. Sugar and food intake in children from four ethnicities**

| The first column refers to sample ID. A- African Americans; B- Burmese; C- Caucasians; H- Hispanics; The numbers in each cell from column B to column F refer to daily consumption of the sugary. Drinks and fast food. 1- means: 1 time/ day; 2- means- more than one time/day |
| --- |
|  |
