# Supplementary material for: Ethnic variation of oral microbiota in children
Source: Sci Rep. 2020 Sep 8;10:14788. doi: 10.1038/s41598-020-71422-y (PMC7478955; doi:10.1038/s41598-020-71422-y)
Supplement: Supplementary file 2 — Supplementary Figure S1. [file 41598_2020_71422_MOESM2_ESM.docx]

**Ethnic Variation of Oral Microbiota in Children**

Thyagaseely S Premaraj^1^, Raven Vella^2^, Jennifer Chung^2^, Qingqi Lin^2^, Panier Hunter^2^, Kori Underwood^1^, Sundaralingam Premaraj^1^, Yanjiao Zhou^2,3^

**Affiliations:**

^1^College of Dentistry, University of Nebraska Medical Center, Lincoln, NE, USA

^2^ Department of Medicine, UCONN Health Center, Connecticut, USA

^3^The Jackson Laboratory for Genomic Medicine, Connecticut, USA

**Corresponding Author:**

Yanjiao Zhou, M.D, Ph.D

Department of Medicine, UCONN Health Center, Connecticut, USA

263 Farmington Ave, Farmington, CT 06030

**Phone: 860-679-6379**

**Email: yazhou@uchc.edu**

**Supplementary Fig.1 Plaque-associated microbiota correlation network.** (a) OTUs that were significantly different in high and low plaque index after adjusting for ethnicity. (b) OTUs that were significantly different in high and low DMFT(t) after adjusting for ethnicity.

d). (c) Correlation was performed to identify the positive and negative correlation between OTUs by SparCC to reveal the co-occurrence network. Each circle represents a single OTU. The size of the circle is proportional to the relative abundance of the OTU. Color of the circle represents positive (r2>0.2, green) or negative (r2<-0.2, red) correlations to plaque index (continues variable). Colors of the lines represents positive (r2>0.4, green) or negative (r2<-0.4, red) correlations between OTUs. OTUs >0.5% is included in the analysis.
